# Hearing in slow-motion: Humans underestimate the speed of moving sounds

**DOI:** 10.1038/srep14054

**Published:** 2015-09-15

**Authors:** Irene Senna, Cesare V. Parise, Marc O. Ernst

**Affiliations:** 1 Cognitive Neuroscience Department and Cognitive Interaction Technology-Center of Excellence, Bielefeld University, Bielefeld, 33615, Germany

## Abstract

Perception can often be described as a statistically optimal inference process whereby noisy and incomplete sensory evidence is combined with prior knowledge about natural scene statistics. Previous evidence has shown that humans tend to underestimate the speed of unreliable moving visual stimuli. This finding has been interpreted in terms of a Bayesian prior favoring low speed, given that in natural visual scenes objects are mostly stationary or slowly-moving. Here we investigated whether an analogous tendency to underestimate speed also occurs in audition: even if the statistics of the visual environment seem to favor low speed, the statistics of the stimuli reaching the individual senses may differ across modalities, hence potentially leading to different priors. Here we observed a systematic bias for underestimating the speed of unreliable moving sounds. This finding suggests the existence of a slow-motion prior in audition, analogous to the one previously found in vision. The nervous system might encode the overall statistics of the world, rather than the specific properties of the signals reaching the individual senses.

To estimate the most likely state of the world despite noisy and incomplete sensory information, the human brain relies on prior knowledge about natural scene statistics^1^. Although this strategy usually results in more precise sensory estimates, it sometimes leads to perceptual illusions. For example, despite its behavioral relevance, visual motion perception is often fundamentally inaccurate, and humans systematically tend to underestimate the speed of moving objects^2^,^3^,^4^,^5^,^6^,^7^,^8^,^9^. This finding has been recently interpreted in terms of a Bayesian prior representing the statistics of speed in natural visual scenes, where static—or slowly moving—signals are more likely to occur^7^,^8^,^9^,^10^. Natural scene statistics, however, might differ across the senses. For instance, the auditory world is a moving world where sounds are only generated by moving objects (though this motion might only involve vibrations in the absence of net motion). This raises the intriguing question of whether an analogous underestimation of speed also occurs in audition as in vision.

To this end, we measured auditory speed perception in a psychophysical task. Given that the influence of Bayesian priors on perceptual estimates scales with the reliability of sensory information, we experimentally manipulated the signal-to-noise ratio of the moving sounds (Fig. 1a). Such a manipulation follows previous studies in visual perception, where the existence of a slow motion prior was inferred by a systematic underestimation of speed when the motion signal was made unreliable, for example by reducing contrast^2^,^3^,^4^,^5^,^6^,^7^,^8^,^10^. If the brain generally assumes that sound sources in the world are usually static, the speed of unreliable auditory signals should also be underestimated, just like in vision. Alternatively, auditory speed perception for unreliable signals might as well be overestimated, possibly due to an auditory prior for fast motion.

## Results

**Figure 1:**
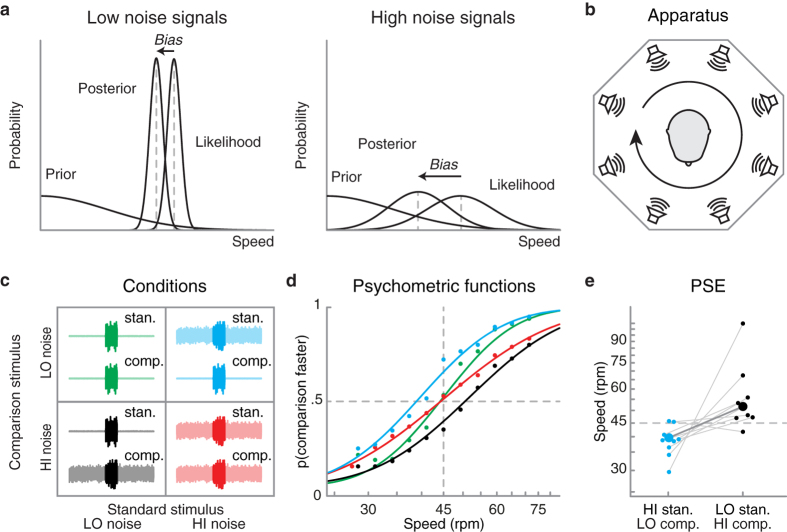
Methods and results. (**a**) Illustration of a Bayesian model accounting for biases in speed perception (see^8^). According to this model, sensory estimates are weighted in proportion to their relative reliability: with reliable sensory estimates (i.e. narrow likelihood function, left panel), a prior for low speed induces only minor perceptual biases (i.e., the mean of the posterior lies close to the mean of the likelihood). However, when sensory information is less reliable (right panel), the same prior induces larger biases toward slow motion. (**b**) Apparatus. A set of eight loudspeakers was arranged on a circle around participants’ head. To generate rotational motion, sounds were cross-faded between neighboring loudspeakers. (**c**) Conditions. Both moving standard and comparison stimuli were played against a background pedestal of pink noise whose intensity was either high (i.e. less reliable information) or low (i.e. more reliable information), for a total of four conditions. In each sub-panel, moving stimuli are graphically represented as saturated waves in the center, while background noise is rendered with lighter colors. (**d**) Psychometric functions for each of the four conditions, obtained by pulling together the data from all participants. Colors represent the different conditions (see panel c, see also Supplementary Fig. S1 for the individual data). The red curve is shallower than the green one, demonstrating that speed perception is less reliable when both standard and comparison stimuli are noisier. The blue and the black curves are shifted apart, indicating that noisier stimuli have to move faster in order to be perceived as fast as less noisy ones. The dashed vertical line represents the speed of the standard stimulus. (**e**) Point of subjective equality (PSE). The PSE is higher when the comparison stimulus is noisier than the standard (black dots), and lower when the standard stimulus is noisier than the comparison (blue dots, see panel c). That is, less reliable auditory stimuli appear to move slower. The dashed horizontal line represents the speed of the standard stimulus. Larger dots represent the PSEs of the aggregate observer (see panel d), while smaller dots represent the individual data. Thin lines connect responses from the same participant.

To psychophysically measure the influence of a potential auditory motion prior, we tested participants in an auditory speed discrimination task. We created a virtual auditory space by arranging a set of eight loudspeakers along a circular array (Fig. 1b). Auditory signals consisted of 1/f noise (pink-noise), which was cross-faded between neighboring speakers to generate moving sounds rotating at different speeds around the participant’s head. Moving stimuli were embedded in 1/f background noise (uncorrelated to the moving sound) simultaneously played by all speakers. In a speed discrimination task, on each trial participants reported which of two consecutive motion stimuli seemed to move faster. One of the stimuli, namely the standard stimulus, moved at a constant speed of 45 revolutions per minute (rpm, corresponding to 270 °/s), while the speed of the comparison stimulus varied on a trial-by-trial fashion. The order of presentation of standard and comparison stimuli pseudo-randomly varied across trials. The duration of the stimuli and their starting position were varied on a trial-by-trial basis (see Methods section), so that they could not be used as ancillary cues for speed.

Given that the influence of prior knowledge on perceptual estimates becomes stronger when sensory information is less reliable^7^,^8^,^9^,^10^ (see also^2^,^3^,^4^,^5^,^6^ for similar findings) (Fig. 1a), we parametrically manipulated the signal-to-noise ratio of the moving sounds. That is, we manipulated the relative intensity of the background noise with respect to the intensity of the moving sound. Each stimulus could present one of two levels of noise (i.e., high or low, see Methods section and Fig. 1c), thus yielding four conditions: two where both standard and comparison had the same level of noise (both high or both low), and two where standard and comparison differed in terms of noise. The first two conditions served to estimate the impact of noise on the precision of perceptual estimates. The other two provided differential information on the influence of priors on auditory speed perception: If indeed there is a prior for lower speeds in audition–as in vision–noisier stimuli should systematically appear to move slower.

We fitted cumulative Gaussian distributions to the proportion of “comparison faster” responses for each condition and participant (11, see Fig. 1d). To confirm that noise consistently reduced the reliability (i.e., precision) of perceptual estimates, we measured the perceptual thresholds (i.e., the just noticeable differences, JND) for each condition (see Methods). As expected, thresholds were lower when both standard and comparison stimuli had a low level of noise (median JND = 4.66 rpm, inter-quartile range = 5.42), as compared to the condition in which both stimuli had a high level of noise (median JND = 7 rpm, inter-quartile range = 14.21, Wilcoxon Signed Rank Test: p = 0.004).

Next, to estimate the relative perceived speed and, hence, the influence of a possible Bayesian prior on perceptual estimates, we calculated the speed at which the standard and the comparison stimuli appeared equally fast (i.e., the point of subjective equality, PSE). Note that the standard stimulus moved at 45 rpm, so if the comparison is perceived as moving at the same speed of the standard, the PSE should be equal to 45 rpm. Results showed that the PSE was higher when the comparison stimulus was noisier than the standard (median PSE = 51.47 rpm, inter-quartile range = 11.66), as compared to the condition in which the comparison was less noisy than the standard (median PSE = 38.86 rpm, inter-quartile range = 5.9, Wilcoxon Signed Rank Test: p = 0.012, Fig. 1d,e). That is, noisier auditory stimuli systematically appear to move slower for eight out of nine participants (Sign test, p = 0.039, Fig. 1e and Fig. S1). The average difference in PSE across the two conditions corresponds to roughly 2 JNDs, that is, two times the threshold for auditory speed discrimination.

## Discussion

The present results demonstrate that human auditory motion perception is fundamentally inaccurate, being characterized by a strong bias for underestimating the speed of unreliable moving sounds. This finding demonstrates the existence of an analogous—though not necessarily the same— prior for low speed in audition as previously reported in vision^7^,^8^,^9^,^10^.

To optimally process those signals that are more frequently encountered, perceptual systems are finely tuned to the statistical properties of the environment (e.g.^12^,^13^,^14^,^15^). Specifically, when estimating a property of the world (i.e., speed), sensory evidence is combined with prior knowledge reflecting the probability distribution of that property in the environment^16^. Thus, in visual motion perception, humans seem to rely on prior expectations based on visual natural scenes statistics, whereby static or slowly moving objects are more likely to occur. However, the statistics of the signals reaching the individual senses might differ between audition and vision. Therefore, if Bayesian priors encode the specific properties of the signals reaching the individual senses, speed perception might be differentially biased in vision and audition. Whether the brain encodes natural scene statistics in a modality independent fashion, or else whether priors differ across sensory modalities is still an open question in sensory neuroscience. The present finding suggest that, at least in the case of speed perception, Bayesian priors seem to represent analogous speed information across the senses, possibly by encoding the statistics of the overall environment rather than the specific properties of the signals reaching the individual senses.

## Methods

### Participants

Eight naïve observers (4 males, mean age = 25.4, SD = 3.1) and C.V.P. took part in the experiment. All participants had normal or correct-to-normal hearing and vision, according to self-report. Participants provided written informed consent and received 6 euros per hour in return for taking part in the study. The experiment consisted of two sessions, each lasting approximately one hour and a half, and taking place on two consecutive days. The study was conducted in accordance to the Declaration of Helsinki, and had ethical approval from the University of Bielefeld ethics committee.

### Apparatus

The experimental setup consisted of eight sound sources arranged along a circle with a diameter of 91.5 cm. Each speaker consisted of two drivers placed at a distance of 32 cm on the vertical axis, mounted on an octagonal frame (Fig. 1b). The center of the speakers was placed approximately at participants’ ear level. Participants sat on a chair placed in the center of the circle and they wore a head-mounted display (z800 3D visor, eMagin, Bellevue, WA). Such a display was used to present the experimental instructions and to otherwise hide the setup from view. Participant’s head was fixed using a helmet mounted on a suspended pole attached to the roof.

### Stimuli and procedure

Moving sounds were generated by cross-fading pink noise stimuli (sampling frequency 44.1 kHz) between neighboring loudspeakers (e.g.^17^). Pink noise was chosen because it provides rich spectral information, allowing participants to rely on both interaural time delay and interaural intensity difference for sound localization, and it is known to occur in a wide variety of natural physical systems (e.g.^18^). Stimulus presentation was controlled by custom software based on the Psychtoolbox 3.0^3^ running on a computer equipped with a M-Audio Delta 1010LT PCI audio card. Sounds were amplified by a Stageline-IMG STA-1508 amplifier, and their average intensity was set at 66 dB SPL. Auditory stimuli were smoothly ramped on and off within a window of 0.5 s. The intensity of the sounds emitted by the 8 speakers was equalized. Moreover, to avoid that residual minor differences in loudness across speakers could be used as cues to solve the experimental task, we slightly jittered the intensity of each speaker on a trial-by-trial basis by multiplying the signals on each channel by a random factor between 0.95 and 1.05.

On each trial, we presented two consecutive sounds, with an inter-stimulus interval of 0.5 s. Each pair of stimuli consisted of a standard and a comparison stimulus, played in randomized order. The standard stimulus had a speed of 45 revolutions per minute (rpm). The comparison stimulus could move at one of eleven different speeds (i.e., 28.38, 31.14, 34.14, 37.44, 41.04, 45, 49.32, 54, 59.34, 65.04, 71.34 rpm). Participants had to judge whether the first or the second sound appeared to move faster by pressing the left or the right mouse button, respectively.

Within each trial, both standard and comparison stimuli moved in the same direction (clockwise or anticlockwise, pseudo-randomly chosen on each trial). In order to prime participants to which direction of motion would occur next, at the beginning of each trial the head mounted display visualized an arrow representing the direction of motion. The arrow appeared at the center of the screen before sound onset and was displayed for the entire duration of the trial. In order to avoid stimulus displacement (e.g., the extent of motion) being used as a proxy for speed (cf.^20^), the duration of each sound was randomly varied between 1.5 s and 2.5 s, including 0.5 s ramps at onset and offset. Likewise, we randomly varied the starting position of each sound at each stimulus presentation (cf.^20^). The range of rotation speeds chosen for the current study was selected based on previous studies (e.g.^21^) and preliminary observations, whereby faster or slower speeds were found to be harder to perceive. After the experiment, all participants verbally reported a clear perception of sounds rotating around them.

The reliability of auditory motion signals was manipulated by changing their signal-to-noise ratio. By comparing the perceived speed of stimuli with different amount of noise, it is possible to infer the existence of a Bayesian prior, given that when sensory information is unreliable (i.e. wider likelihood function), the brain relies more heavily on prior knowledge (Fig. 1a; see also^4^,^5^,^6^,^7^,^8^,^9^,^10^ for a similar manipulation in visual speed perception). To change the signal-to-noise ratio, we added a pedestal of pink noise to the moving stimuli. This pedestal was played simultaneously by all loudspeakers. Given that the background noise was simultaneously played by all loudspeakers in the circular setup, the rendered sound is physically compatible with a circular acoustic source rotating around the observer at any possible speeds (from zero to infinity). That is, the speed information provided by the background noise is completely ambiguous, and it can be represented by a flat likelihood function over speed (see Supplementary Fig. S2 for further details). Therefore, when coupled with the moving stimulus signal, such a background noise just made the overall auditory motion signal less reliable, without introducing any additional speed information. Both standard and comparison stimuli could have one of two levels of pedestal noise, high (60 dB SPL) or low (42 dB SPL). These levels of noise were chosen based on preliminary observations, where we made sure that the added noise was sufficient to differentially modulate the reliability of perceptual estimates, without fully masking the moving stimuli. The intensity of the stimuli was adjusted, so that stimuli and pedestal noise together were equalized in loudness: when pedestal noise was higher, stimulus intensity was lower (64.7 db SPL); when pedestal noise was lower, stimulus intensity was higher (~66 db SPL). Thus, all stimuli (i.e., the moving signal plus the background noise) were played at the same overall intensity (66 dB SPL). The background noise in the room, in the absence of any stimulus, was 41 dB SPL. The experimental design consisted of four conditions, presented randomly intermixed, differing in terms of the intensity of the pedestal noise in both the standard and the comparison stimuli: (1) low noise standard – low noise comparison (Fig. 1c, top-left), (2) high noise standard – high noise comparison (Fig. 1c, bottom-right), (3) low noise standard – high noise comparison (Fig. 1c, bottom-left), (4) high noise standard – low noise comparison (Fig. 1c, top-right). Overall, the experiment consisted of 880 trials, that is, 220 trials per condition.

To motivate participants, every 22 trials the head-mounted display showed the percentage of correct responses, and participants could take a break. Given that the feedback was blocked, it did not provide information about the performance on a trial-by-trial basis, and therefore it could not be used for task learning. Before the first experimental session, participant underwent a training session to familiarize with the task and the stimuli. The training consisted of one block of 176 stimuli. An auditory feedback (a beep) was delivered during training whenever participant’s produced an incorrect response. Only low noise standard – low noise comparison (Fig. 1c, top-left), and high noise standard – high noise comparison combinations (Fig. 1c, bottom-right) were presented during the training session.

### Data analysis

For each condition and participant, we calculated psychometric functions by fitting cumulative Gaussian distributions to the proportion of “comparison faster” responses^8^. In order to reduce the number of parameters, we fixed the PSE of the low noise standard - low noise comparison, and high noise standard - high noise comparison conditions at the standard speed (45 rpm). Goodness of fit values for each curve and participant are reported in Supplementary Table S1.

We then calculated the perceptual thresholds (i.e., just noticeable differences, JNDs) for each condition from the psychometric curves by halving the difference between the speed at which participants made 25% and 75% “comparison faster” responses. While perceptual threshold in the condition in which both comparison and standard stimuli had a higher level of noise differed from the one in which both stimuli had a lower level of noise (see Results and Discussion section above), the JNDs in the other two conditions did not significantly differ from each other (high noise standard–low noise comparison = 4.81 rpm, inter-quartile range = 4.2 rpm; low noise standard–high noise comparison = 4.84 rpm, inter-quartile range = 15.66 rpm, Wilcoxon Signed Rank Test: p = 0.1).

The point of subjective equality (PSE), specifying the speed at which the standard and the comparison stimuli appeared equally fast, was calculated for each subject and each condition as the speed required to respond ‘‘comparison faster’’ half of the times. Analyses were performed on the individual data from each participant, while the curves in Fig. 1d are fitted to the mean-aggregated data, and shown for illustrative purposes only.

## Additional Information

**How to cite this article**: Senna, I. *et al.* Hearing in slow-motion: Humans underestimate the speed of moving sounds. *Sci. Rep.* **5**, 14054; doi: 10.1038/srep14054 (2015).

## Supplementary Material

Supplementary Information
