# Supplementary material for: Hearing in slow-motion: Humans underestimate the speed of moving sounds
Source: Sci Rep. 2015 Sep 15;5:14054. doi: 10.1038/srep14054 (PMC4570192; doi:10.1038/srep14054)
Supplement: Supplementary Information [file srep14054-s1.pdf]

Supplementary Information

Hearing in slow-motion: Humans underestimate the speed of moving sounds.

Irene Senna, Cesare V. Parise, Marc O. Ernst

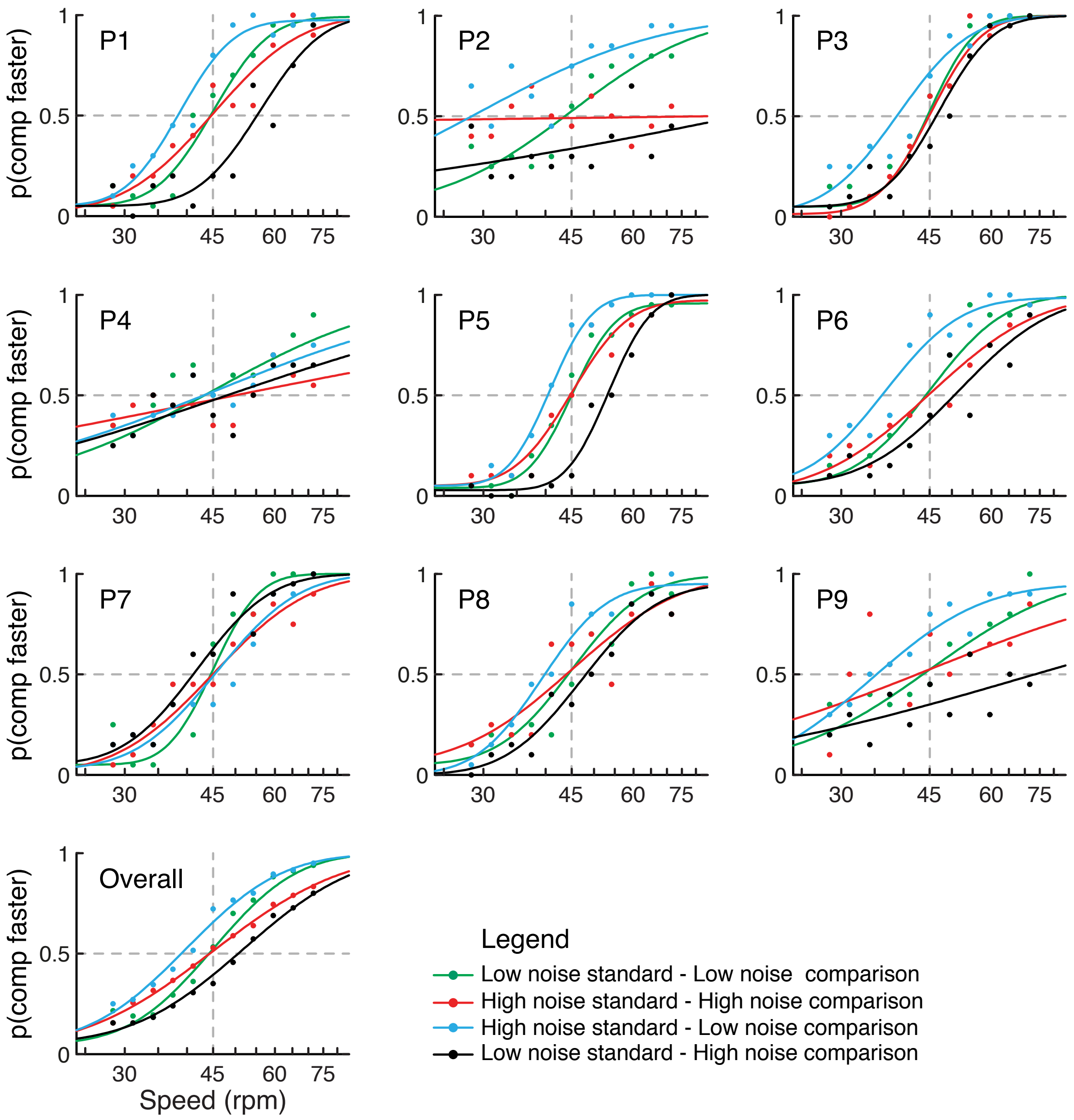

Supplementary Figure S1. Psychometric functions for each participant and for the aggregate observer (bottom, see also Figure 1d), obtained by pulling together the data from all participants. Analyses were performed on the individual data from each participant, while aggregated data are shown for illustrative purposes only. Colors represent the different conditions. For eight out of nine participants, the noisy stimuli (black lines) appear to move slower than the more reliable ones (blue lines). The dashed vertical line represents the speed of the standard stimulus.

Participants	Deviance			
	LO n. stand.<br>LO n. comp.	HI n. stand.<br>HI n. comp.	HI n. stand.<br>LO n. comp.	LO n. stand.<br>HI n. comp.
P1	9.09	11.68	9.97	18.96
P2	8	6.78	12.82	13.03
P3	10.57	9.98	12.14	8.41
P4	9.46	6.40	3.85	8.8
P5	6.09	9.77	3.85	7.93
P6	11.76	6.50	12.02	11.66
P7	19.37	6.73	11.13	9.05
P8	13.57	17.74	7.80	7.90
P9	11.96	27.69	4.03	9.73

Supplementary Table S1. Goodness of fit (i.e. deviance, see[11]) for each curve, fitted for each condition, and participant.

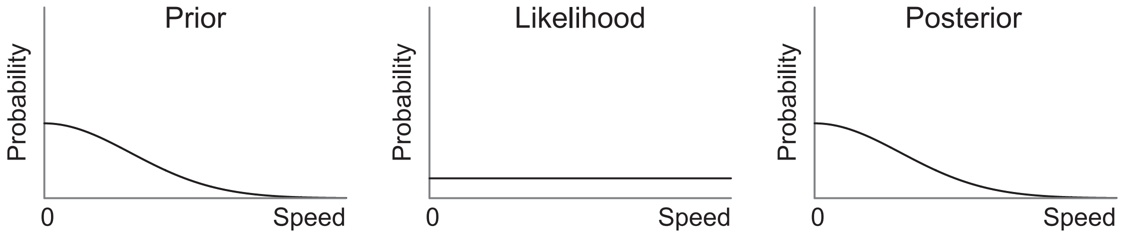

Supplementary Figure S2. Manipulation of the signal-to-noise ratio. The pedestal added to the moving stimuli, and played simultaneously by all loudspeakers in a circular setup, is physically compatible to a circular acoustic source rotating around the observer at any possible speeds, and can be represented by a flat likelihood function with uniform probability over all possible speeds (central panel). While such a background noise (i.e. the likelihood function) does not introduce any additional speed information to the

moving signal, when played alone it is usually perceived (i.e. the posterior) as static (or slowly moving). Indeed, when the flat likelihood of a fully ambiguous stimulus is combined with a prior for low speed (left panel), the stimulus will be subjectively perceived as static (i.e. the combination of flat likelihood with an informative prior will lead to a posterior that correspond to the prior; right panel). In other words, adding such a background noise to the moving signals reduces the reliability of motion perception (see Results, higher JND for higher background noise), without adding any additional speed information.
